# Soldiers at the London Hospital: The Method and Procedure Adopted

**Published:** 1915-03-20

**Authors:** 


### SOLDIERS AT THE LONDON HOSPITAL.

### The Method and Procedure Adopted.

(From a Correspondent.)

The majority of the voluntary hospitals have by this time made arrangements to receive sick and wounded soldiers. It may, therefore, interest readers of The Hospital to hear something of the method which has been adopted at the London Hospital to deal with these patients.

Immediately after the declaration of war, the Board of the hospital offered the use of 500 beds, 250 for soldiers and 250 for sailors. Although this offer was at once accepted, it was some weeks before any patients were sent to the hospital, but suddenly, on a Sunday morning early in September, a message was received from the War Office asking whether 100 wounded men from the Front could be accommodated that evening. A response in the affirmative was immediately sent, and later in the day, as The Hospital recorded at the time, a second message came, requesting the hospital authorities to undertake the transport of these wounded men from Waterloo Station.

### The Military Superintendent.

Mr. E. W. Morris, the secretary, and Mr. Elliot, assistant secretary, assisted by the resident medical officers and nursing staff, made all necessary arrangements for the reception of these patients. Captain Fenwick, F.R.C.S., one of the consulting surgeons, was appointed by the War Office Military Superintendent.

This first contingent of wounded men arrived late in the evening, and, thanks to the arrangements which had been made, they were very soon comfortably settled in their beds. While these soldiers were being received, a further urgent request came from the War Office asking that accommodation be provided for a second contingent of 200 men. These arrived at the hospital early the following morning.

### The New Special Staff.

From that day until the present time over 2,200 soldiers have been received and treated in the wards. In order to deal efficiently with such large numbers of patients as these it has been necessary to organise a special staff, who work under the general control of Captain Fenwick. The work has been carried out in a most satisfactory manner and has earned the full approval of the War Office authorities.

The method of procedure with regard to these patients is as follows. A notification of the number of vacant beds available for soldiers is forwarded each morning to the Commanding Officer of the Queen Alexandra Military Hospital, Millbank, with which institution the London Hospital is affiliated, and the military authorities then notify the hospital of the number of new patients which may be expected to arrive that evening. Information is also given as to the number of stretcher and walking cases respectively, the ambulances and motor-cars for transport being provided by the military authorities.

Before the arrival of a contingent a separate card has been prepared for each vacant bed, showing the ward and number of bed allotted to each soldier. To avoid any delay a series of coloured cards has been devised, each colour representing a different ward (medical, surgical, etc.), so that Mr. Morris, who receives all the patients, on asking the nature of each case, can allot it at once to its proper ward from the selection of available beds which is apparent from the coloured cards in his possession. The men are thus transferred at once from the ambulance to the beds provided for them.

### Clothing, Dental Treatment, and Railway Passes.

Beef-tea or other suitable refreshments are served to the men on their arrival. The clothing of each is placed at once in a separate canvas bag, labelled with his name, number of bed, etc., and is taken charge of by a porter, who is responsible for seeing that the clothing is thoroughly sterilised and cleaned as may be found necessary. Special difficulty arises from the sheepskin coats which many of the men are wearing, for they require very careful and thorough disinfection, being often full of vermin.

As many of the soldiers arrive with insufficient or worn-out clothing, a supply of uniforms, underwear, boots, etc., has been provided by the military authorities, and is distributed as found necessary.

Within a few hours of the arrival of any contingent, a complete list of names, numbers, regiments, etc., of the soldiers is prepared and forwarded to the military authorities at Millbank.

While in the wards these patients are entirely under the care of the medical and surgical staff of the hospital, who are solely responsible for treatment. Mention should be made of the special arrangements made by the military authorities for dental treatment where necessary, which is carried out by the hospital dental surgeons, the cost being defrayed by the War Office.

When the patients are sufficiently recovered they are drafted, with the consent of the military authorities, to various convalescent homes in the country, which have been generously placed at the disposal of the hospital for this purpose. When ready for final discharge the soldiers are sent direct to the depôts of their respective regiments, the hospital being authorised by the War Office to issue the necessary railway passes.

In order to provide amusement and occupation for the soldiers during their stay in the wards an ample supply of books, games, etc., has been given by many friends, and much interest has been aroused by various competitions for prizes for the best models of the trenches made in modelling-clay, and also for trestle bridges and other military appliances made from pieces of wood, wire, etc. Many of these models have been seen by Army officers, who have expressed admiration of their correctness and workmanship.

